# Altered Signal Transduction in the Immune Response to Influenza Virus and *S. pneumoniae* or *S. aureus* Co-Infections

**DOI:** 10.3390/ijms22115486

**Published:** 2021-05-22

**Authors:** Janine J. Wilden, Jasmin C. Jacob, Christina Ehrhardt, Stephan Ludwig, Yvonne Boergeling

**Affiliations:** 1 Institute of Virology Muenster (IVM), Westfaelische Wilhelms-University Muenster, 48149 Muenster, Germany; janine.wilden@wwu.de (J.J.W.); Jasmincarina.Jacob@ukmuenster.de (J.C.J.); ludwigs@uni-muenster.de (S.L.); 2 CiM-IMPRS, The Joined Graduate School of the Cells in Motion Interfaculty Centre, University of Muenster and the International Max Planck Research School—Molecular Biomedicine, 48149 Muenster, Germany; 3 Section of Experimental Virology, Center for Molecular Biomedicine (CMB), Institute of Medical Microbiology, Jena University Hospital, 07745 Jena, Germany; christina.ehrhardt@med.uni-jena.de; 4 “Cells in Motion Interfaculty Center (CIMIC)”, WWU Muenster, 48149 Muenster, Germany

**Keywords:** influenza virus, *S. pneumoniae*, *S. aureus*, immune response

## Abstract

Influenza virus is a well-known respiratory pathogen, which still leads to many severe pulmonary infections in the human population every year. Morbidity and mortality rates are further increased if virus infection coincides with co-infections or superinfections caused by bacteria such as *Streptococcus pneumoniae* (*S. pneumoniae*) and *Staphylococcus aureus* (*S. aureus*). This enhanced pathogenicity is due to complex interactions between the different pathogens and the host and its immune system and is mainly governed by altered intracellular signaling processes. In this review, we summarize the recent findings regarding the innate and adaptive immune responses during co-infection with influenza virus and *S. pneumoniae* or *S. aureus*, describing the signaling pathways involved and how these interactions influence disease outcomes.

## 1. Introduction

Influenza viruses cause highly infectious lung diseases and infect hundreds of thousands of people every year worldwide. Illnesses with seasonal influenza viruses range from mild to severe cases, whereas high-risk groups with pre-existing lung diseases are more predisposed to hospitalization and even death. Of an estimated three to five million severe cases, about 290,000 to 650,000 patients die due to respiratory failure [1,2].

Secondary pulmonary infections caused by influenza viruses in combination with bacteria lead to challenging problems in clinics, including increased numbers of fatal cases. It has been known for a long time that influenza viruses pave the way for secondary bacterial infections, e.g., by increasing the exposure of surface receptors on epithelial cells to facilitate bacterial adherence or damaging the epithelial barrier to enable bacterial invasion into deeper tissues [3,4]. In these cases, clinicians are confronted with severe lung damage induced by a dysregulated immune response, which is caused by misdirected signal transduction [5,6]. During previous influenza pandemics from 1918–2009, and also during seasonal outbreaks, many influenza-related deaths result from secondary bacterial infections [7,8,9].

Approximately 30–40% of hospitalized patients diagnosed with influenza suffer from acute pneumonia due to influenza virus infections alone or in conjunction with secondary bacterial infections, primarily caused by *S. pneumoniae* or *S. aureus* [7,10]. These bacteria are also included in the WHO’s priority pathogen list, with medium priority for *S. pneumoniae* and high priority for *S. aureus,* highlighting them as threats to public health and encouraging research to identify possible targets for treatment [11]. Influenza virus infections with subsequent *S. pneumoniae* or *S. aureus* infections are detectable worldwide [8,12] and the situation may become even more critical with the increasing emergence of resistant bacterial strains. Influenza virus infections in conjunction with methicillin-resistant *S. aureus* (MRSA) strains are reported to induce higher mortality compared to non-resistant *S. aureus* [13] or *S. pneumoniae* co-infections, and anti-bacterial treatment in early stages of influenza virus infection is critical to reducing the risk of severe outcomes [14]. Additionally, around 30% of the human population are nasal carriers of *S. aureus* without any symptoms of a bacterial infection [15], which increases the occurrence of co-infections in general. *S. pneumoniae* is also able to colonize transiently and asymptomatically in immunocompetent hosts [16]. Even though bacterial colonization with pathogenic strains in most cases remains undetected because they have become part of the commensal microbiota, a trigger such as a viral infection can induce severe health consequences [17].

Interestingly, co-infection scenarios of influenza viruses and *S. pneumoniae* or *S. aureus* differ in the sequence of infection. Although *S. pneumoniae* is often reported to cause severe bacterial infections after viral clearance when the host is more susceptible [18,19,20], co-infections with *S. aureus* are more likely to occur concomitantly [21,22]. This disparity can be attributed to influenza virus-induced secretion of various cytokines and chemokines, which differently affect the infectivity of the respective bacteria and thereby also the outcome of bacterial co-infections [23]. Numerous complex interactions between the pathogens and the host determine the severity of co-infections, in which the deregulation of the immune response through dysregulated signal transduction plays a major role. Overreactions of the immune response due to hyper-activated or inhibited intracellular signaling pathways lead to damage of the lung epithelial cell barrier, altered immune responses and increased inflammatory reactions [24,25,26].

In this review, we summarize the latest scientific results regarding co-infections with influenza viruses and *S. pneumoniae* or *S. aureus*, including essential changes in the innate and adaptive immune responses, mediated by the mutual impact of the pathogens on molecular signal transduction pathways.

## 2. Immune Response in Co-Infections

The immune system is composed of an innate and an adaptive response and has evolved to protect the host from diseases. The innate immune system mediates the undirected immune response and is mostly composed of a group of proteins (such as opsonins of the complement system and enzymes such as defensins), natural killer (NK) cells and phagocytic cells (monocytes, neutrophils and macrophages) that recognize conserved structures of the invaders [27]. The adaptive immune system represents the specific immune response of the host against a particular antigen. Two different types of lymphocytes, namely T and B cells, mediate this kind of immunity. T cells are involved in cell-mediated adaptive immune responses, whereas B cells are responsible for the production of antigen-specific antibodies [28,29]. As described above, influenza virus primes the host for secondary bacterial infection and *vice versa*, affecting disease outcomes. How the pathogens circumvent and modulate the different parts of the hosts’ immune response will be reviewed in detail in the following sections.

### 2.1. Innate Immunity

#### 2.1.1. Interference of Co-Infecting Pathogens with the Complement System

**Figure 1:**
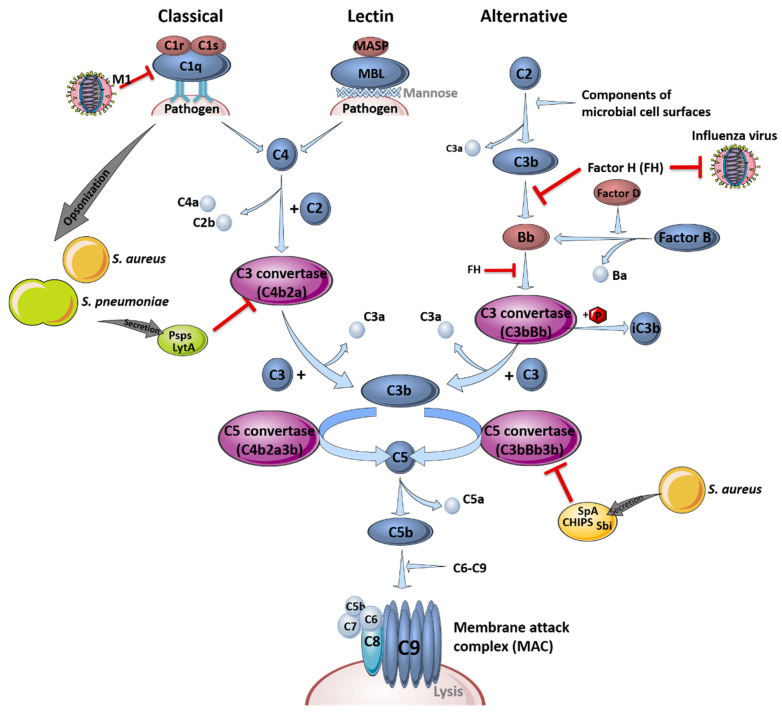
The complement system and its role in pathogen clearance. Schematic overview of the activation of the classical, the lectin and the alternative signaling pathway, leading to the formation of the membrane attack complex (MAC). Interference of the different pathogens with the complement system pathways is indicated by red inhibition arrows. This figure was created using Servier Medical Art templates, which are licensed under a Creative Commons Attribution 3.0 Unported License; https://smart.servier.com (accessed on 11 February 2021).

The complement system is part of the first line of defense acting against infections and is critical for pathogen clearance. In general, it is comprised of three different pathways, the classical, the lectin and the alternative pathway, which are either receptor-dependently or independently activated. Different plasma proteins, so-called complement factors (C1-9), bind to the surface of pathogenic invaders to facilitate immune cell detection to enable phagocytosis, induce inflammation processes or support pathogen clearance [30]. A schematic overview of the different complement system pathways and their interference with influenza virus or bacterial components during infections is depicted in Figure 1.

The complement system recognizes the aforementioned pathogens in the early stages of infection in the upper and lower respiratory tract and bridges the innate and adaptive immune response [31]. Signal transduction is induced via specific cleavage of the complement factors C5 and C3, which serve as chemoattractants and recruit immune cells to sites of activation by binding to their anaphylatoxin receptors C5aR (CD88) and C3aR, respectively. Factor H (FH), an inhibitor of the alternative pathway to prevent hyper-induction by limiting the formation of C3 convertase, is able to block influenza viruses, limiting virus entry by impeding the action of the viral surface proteins hemagglutinin (HA) and neuraminidase (NA) [32]. *S. pneumoniae* and *S. aureus* are mainly opsonized by complement factor C3b or C4b, which improves the recognition by phagocytic cells or resulting in the formation of the membrane attack complex, causing lysis of the bacteria by forming a pore [33].

However, influenza viruses and bacteria also developed particular mechanisms to evade the complement system. For example, FH-mediated inhibition of viral entry is only effective against H1N1 influenza virus strains. Interestingly, the otherwise restrictive effects on viral entry are conversely promoting H3N2 entry, which interacts less efficiently with FH [32]. In contrast, not all manipulations of the complement system by influenza viruses result in immune evasion. For instance, influenza virus interferes with the complement decay-accelerating factor (DAF/CD55), which usually induces protective signaling cascades against unspecific complement attacks to prevent an increased inflammatory response. Binding and cleavage of DAF/CD55 by viral proteins HA and NA results in an overreaction of the complement system and leads to a massive recruitment of innate immune cells. Here, overshooting secretion of cytokines and chemokines induces tissue damage and facilitates the invasion of e.g., *S. pneumoniae* or *S. aureus* without affecting viral load [31].

In addition, bacteria such as *S. pneumoniae* have established mechanisms to affect and evade the complement system. The structure of the polysaccharide capsule impedes the adherence of complement factors to the bacteria, which reduces opsonization and the activation of complement-associated signaling pathways [34]. In addition, opsonization is reduced through competitive binding of the pneumococcal surface protein A (PspA) to phosphocholine in the pneumococcal wall [35]. PspA, pneumococcal surface protein C (PspC), as well as the cell wall amidase LytA [36], were shown to prevent the formation of C3 convertase, a serine protease cleaving peptide bonds on the surface of pathogens, by binding of complement regulator C4-binding protein (C4BP) [37]. Furthermore, LytA hampers the recognition of phosphocholine by the major acute phase protein C-reactive protein and is able to interact with FH [36]. Accumulation of FH reduces C3b deposition on the bacterial surface. Additionally, LytA is capable to directly degrade already deposited C3b and inactive C3b, which additionally reduces the amount of surface-bound C3b. This effect was shown to be independent of capsule presence [36]. In a transtympanic-inoculation-induced mouse model of acute pneumococcal otitis media, it was reported that a preceding influenza virus infection increased the bacterial burden in the ear, whereupon the complement system was identified as a critical key player [38]. In that study, a reduction in C3a and C5a levels in serum and middle ear lavage samples was observed. Transferring this result to a co-infection scenario exclusively in the lung, the impaired opsonization of bacterial pathogens, as well as the decreased activation of immune cells due to the lack of specific complement system-mediated signal transductions, could be hypothesized. In terms of *S. aureus* infection, the mechanisms of opsonization and activation of the complement system are comparable to those of *S. pneumoniae*. Furthermore, *S. aureus* has evolved several defense mechanisms regulated by the accessory gene regulator (*agr)*, which induces the secretion of different proteins such as proteases. Bacterial proteases can cleave the complement factor C3 to avoid opsonization, thereby inhibiting the induction of complement system signaling and, thus, immune cell activation. Secreted proteins such as Sbi (staphylococcal binder of immunoglobulins), SpA (staphylococcal protein A) and CHIPS (chemotaxis inhibitory protein of *S. aureus*) are able to bind and block immunoglobulin G (IgG) and C3 or C5a, resulting in an impaired recruitment of neutrophils, which improves the infection efficiency of influenza viruses [39,40].

In summary, influenza viruses and *S. pneumoniae* or *S. aureus* are able to interfere with the signal transduction of the complement system, promoting viral and bacterial co-infection by blocking specific complement system key players. Compared to *S. pneumoniae* infections, which often occur after viral clearance, the suppression of the complement system in *S. aureus* co-infection could lead to increased complications, since viral clearance is delayed.

#### 2.1.2. Alterations in Cell Intrinsic Signaling Affect the Secretion of Cytokines and Chemokines in Innate Immunity

In addition to the complement system, intra- and extracellular detection of viruses and bacteria mediates the activation of the innate immune response by inducing cellular signaling cascades, resulting in the production of a variety of cytokines and chemokines [41].

In general, highly conserved small molecule motifs, so-called pathogen-associated molecular patterns (PAMPs), are recognized in different cell types via so-called pattern recognition receptors (PRRs). Toll-like receptors (TLRs), retinoic acid-inducible gene I (RIG-I)-like receptors (RLRs) and nucleotide oligomerization domain (NOD)-like receptors (NLRs) activate downstream transcription factors such as interferon (IFN) regulatory factors (IRFs) and the nuclear factor ‘kappa-light-chain-enhancer’ of activated B cells to induce IFNs and other cytokines and chemokines [41]. TLR3 and RIG-I are the two major PRRs that detect influenza virus infections. TLR3 senses double-stranded ribonucleic acid (dsRNA) in endosomes [42], whereas RIG-I binds to 5′-triphosphorylated single-stranded RNA (ssRNA) in the cytosol [43]. In addition to the rarely characterized recognition of viral ssRNA by NOD2 [44], TLR7 has also been described as an endosomal receptor that recognizes ssRNA of influenza viruses. However, in contrast to the other aforementioned PRRs, stimulation of TLR7 alone did not have any effect on bacterial clearance in co-infection scenarios [45]. Gram-positive bacteria such as *S. pneumoniae* and *S. aureus* are mainly detected extracellularly via TLR2 through the recognition of lipoteichoic acid in the bacterial cell wall [46]. Since *S. aureus* is able to survive intracellularly, recognition by NOD2 was also reported [47].

**Figure 2:**
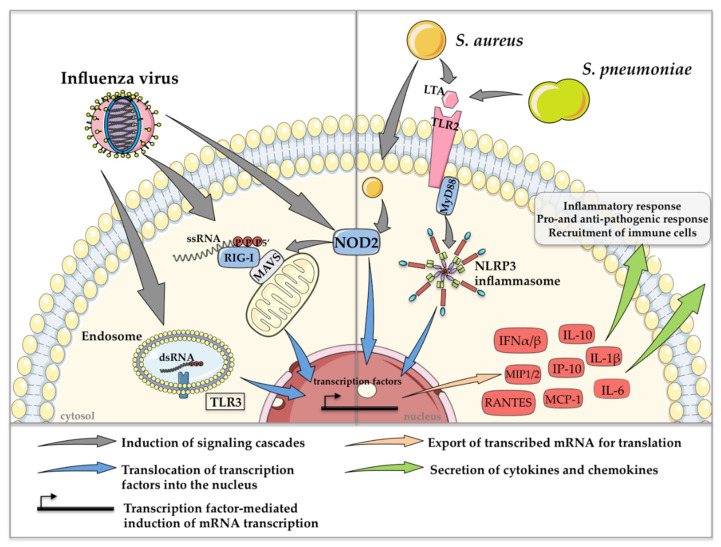
Schematic depiction of the most prominent PRR-mediated signaling events in co-infections with influenza viruses and *S. pneumoniae* or *S. aureus*. Detection of extra- and intracellular PAMPs induces the expression of type I IFN and various cytokines and chemokines, resulting in pro- and anti-pathogen responses and the recruitment of immune cells. This figure was created using Servier Medical Art templates, which are licensed under a Creative Commons Attribution 3.0 Unported License; https://smart.servier.com (accessed on 11 February 2021).

The most prominent PRR-mediated signaling mechanisms, which are induced during co-infection with influenza viruses and *S. pneumoniae* or *S. aureus*, resulting in the regulation of cytokine and chemokine expression, are depicted in Figure 2.

Cytokines such as IFNs and chemokines, which are secreted after recognition of influenza viruses and *S. pneumoniae* or *S. aureus* play an essential role in pathogen clearance [48]. Interestingly, pre-activation of TLR3 and RIG-I via the synthetic dsRNA analog poly I:C before infection with *S. pneumoniae* or MRSA resulted in an enhanced bacterial burden in the lungs of mice, accompanied by extensive areas of consolidation and inflammation, indicating that viral PRR activation is a trigger for increased susceptibility to secondary bacterial infection [45]. In this context, the abolishment of IFN-α/β receptor (IFNAR) signaling via antibody treatment restored bacterial clearance after poly I:C treatment, suggesting that secondary *S. pneumoniae* or MRSA infection might benefit from enhanced type I IFN production induced by influenza virus infection [45]. Likewise, poly I:C-treated *IFNAR1*^−/−^ animals showed reduced lung damage compared to poly I:C-treated wild-type (WT) mice after three days of bacterial infection [45]. Another example of the bacteria-supportive impact of type I IFNs in co-infections is the induction of the anti-inflammatory cytokine interleukin-10 (IL-10), which in general is induced in order to keep inflammations under control. However, co-infected mice show a significant increase in IL-10 concentrations in the lungs, which is accompanied by an increased bacterial burden compared to only influenza virus- or *S. pneumoniae*-infected control mice. Strikingly, mice that were pretreated with neutralizing monoclonal antibodies against IL-10 before secondary pneumococcal infection showed significantly reduced bacterial burdens and increased survival [20]. The same effects are described for co-infection scenarios with influenza virus and *S. aureus* [49]. In that study, the impairment of neutrophil function was directly correlated with increased IL-10 levels. In conclusion, type I IFN expression induces high amounts of IL-10, which plays a role in priming the host for secondary *S. pneumoniae* and *S. aureus* infection after influenza clearance [20,49,50].

IFN induction and signaling can also be affected by mitogen-activated protein kinases (MAPKs) [51]. The activation of MAPK p38 in influenza virus single [52] or co-infections with *S. pneumoniae* [53] or *S. aureus* [54] was shown to play an essential role in the production of cytokines, such as IFNβ, IL-1β, tumor necrosis factor α (TNFα) and IL-6, in vitro and in vivo [54]. IL-6, an important pro- and anti-inflammatory cytokine [55], was described to be significantly upregulated in influenza virus and *S. pneumoniae* [56] as well as *S. aureus* co-infections [54,57]. Surprisingly, a recent study demonstrated that IL-6 is a protective agent in *S. pneumoniae* co-infection, since its depletion led to significantly increased bacterial burdens in the nasal lavage, heart, blood, spleen and liver in mice. This observation was in line with the finding that *Il-6*^−/−^ ^−/−^mice died earlier compared to WT mice in co-infections. The protective actions of IL-6 were related to the mediation of cell homeostasis, as well as phagocytosis [56]. So far, this protective action of IL-6 has not been described for co-infection scenarios with *S. aureus*.

In general, the elevated induction of pro-inflammatory cytokines and chemokines, such as IL-6, TNFα, IL-1β, IL-8 and monocyte chemoattractant protein 1 (MCP-1), among others, have been correlated to the induction of a so-called ‘cytokine storm’, leading to a massive inflammatory immune response [58]. This uncontrolled release of pro-inflammatory cytokines and chemokines, which is accompanied by severe lung injury, is nowadays considered a possible therapeutic target [59,60,61]. As mentioned earlier, *S. pneumoniae* develops its main pathogenic potential after viral clearance, whereas *S. aureus* already benefits from synergistic effects during acute influenza virus infection. In influenza virus and *S. aureus* co-infections, this synergistic effect is supported by increased activation of bacterial superantigens such as enterotoxin B [62] and toxic shock syndrome toxin 1 (TSST-1) [63]. This leads to the uncontrolled activity of T cells and an enormous secretion of cytokines such as TNFα and IFN-γ [64], resulting in an increase in the inflammatory potential during co-infection. Moreover, an overreaction of monocytes and the vast production of chemokines is provoked, which results in the massive recruitment of macrophages into the lungs [65], induced by elevated levels of MCP-1, macrophage inflammatory proteins 1 and 2 and IFN-inducible protein 10 (IP-10) [66,67]. Furthermore, influenza virus infection promotes the production of cytokines and chemokines that are responsible for neutrophil recruitment. Interestingly, in the presence of *S. aureus* strains expressing the β-pore-forming toxin Panton-Valentine leukocidin (PVL), recruited neutrophils are destroyed and release proteases that further damage the airway epithelium [68]. These effects of influenza viruses on bacterial toxins have not been described for *S. pneumoniae* to date. Moreover, the NOD-leucin rich repeat- and pyrin domain-containing protein 3 (NLRP3) inflammasome also contributes to increased pro-inflammatory cytokine levels after the detection of *S. pneumoniae* or *S. aureus*. This activation occurs in a myeloid differentiation primary response 88 (MyD88)-dependent manner, which leads to an exaggerated induction of the immune response [69].

A summary of the functions of the different cytokines discussed can be found in Table 1.

In summary, signal transduction induced after the detection of influenza virus and *S. pneumoniae* or *S. aureus* by their respective cellular PRRs results in the expression of different cytokines and chemokines (e.g., IL-10 and IL-6). In the context of co-infections, pathogen clearance is influenced in various ways and can result in the induction of a cytokine storm. Although influenza virus infection in general has shown bacteria-supporting properties, *S. aureus* might possess more pathogenic potential compared to *S. pneumoniae* due to the simultaneous infection with influenza viruses, causing a potentiation of the toxicity of bacterial virulence factors by the virus [21,22]. This would make *S. aureus* a potentially more serious pathogen in co-infections with influenza viruses.

#### 2.1.3. Recruitment and Activation of Innate Immune Cells

##### Macrophages

Alveolar macrophages (AMs) are among the most abundant cell types found in the respiratory lumen and play a crucial role in innate immunity against viral and bacterial infection [74,75]. In recent years, several studies have indicated an impaired anti-bacterial activity of AMs after a preceding influenza virus infection, which could be attributed to an influenza virus-mediated modulation of the activity of different signaling cascades, such as the IFN-induced JAK (Janus kinase)-STAT (signal transducer and activator of transcription) pathway [76,77,78]. It was shown that co-infection with influenza virus and *S. aureus* seems to alter the composition of macrophage subtypes in mouse lungs via modulation of STAT2 signaling [79]. *Stat2*-deficient mice, which were co-infected with MRSA six days after influenza virus infection, displayed a significantly increased frequency of M1/M2 dual phenotype macrophages. M1-activated macrophages show an enhanced microbicidal capacity and the secretion of pro-inflammatory cytokines, whereas M2-activated macrophages resolve inflammation and support tissue healing through the activation of various signaling cascades via the expression of TLR ligands or ILs [80]. Even though the level of M1 macrophages was also elevated, an overall increase in frequency was considerably more prominent in the case of M1/M2 macrophages. Surprisingly, this dual phenotype was associated with improved bacterial clearance in influenza virus and MRSA co-infected *Stat2*^−/−^ mice, indicating the bacteria-supportive influence of STAT2 signaling via the suppression of macrophage activation [79]. The impaired anti-bacterial function of AMs during co-infection was further characterized by an increase in major histocompatibility complex class II (MHCII) expression. In conclusion, these macrophages rather seem to support the adaptive immunity against the preceding influenza virus infection and are therefore not efficient in setting up the optimal innate immune response against the secondary *S. pneumoniae* infection [74]. This described effect was connected to influenza virus-induced IFNγ secretion [74]. Furthermore, inhibition of NK cell-dependent TNFα production results in decreased AM phagocytic activity and reduced *S. aureus* clearance [73], demonstrating the altered anti-bacterial activity of AMs during co-infection. There are several theories as to the ways in which a preceding influenza virus infection can interfere with macrophage responses to secondary bacterial infection. However, the specific triggers leading to changes in immune cell recruitment via the modulation of different signal transduction processes are not yet fully understood and remain controversial. It has been reported that influenza virus infections result in a significant depletion of AMs in the lungs due to cell death one week after infection *in vivo* [76,78,81]. Therefore, AM-mediated early bacterial clearance is inhibited, which opens up a time window for increased susceptibility to secondary pneumococcal infection [76]. On the contrary, another study claims that after one week of virus infection, the number of resident macrophages is comparable to that of uninfected animals. However, rather than the number, the phagocytic activity of resident AMs as well as of newly recruited macrophages seems to be reduced by viral infections [74]. This was recently confirmed by another research group, who stated that a preceding viral infection affects the anti-bacterial function of AMs, which verifies the aforementioned points [82]. Notably, it was reported that the mouse strain used in these studies may influence the effect of influenza virus infection on AMs based on an IFNγ-dependent reduction of AM levels in BALB/c mice, which could not be observed in C57BL/6 mice [77].

##### Neutrophils

In addition to AMs, neutrophils are important key players in innate immunity and are described to be the first responders to infections [83]. Today, it is known that a preceding influenza virus infection hampers the neutrophil response to secondary bacterial infection in regard to neutrophil recruitment, phagocytic activity and intracellular killing. In terms of T cell-dependent neutrophil recruitment in *S. aureus* and influenza virus co-infection, it was shown *in vivo* that the influenza virus-induced immune response led to an inhibition of the cellular signaling needed for IL-17 production, which is also essential for bacterial clearance [84]. The exact mechanism is discussed in Section 2.2.1. However, not only the amounts of recruited neutrophils, but also the phagocytic activity and intracellular killing ability are decisive for bacterial clearance. Neutrophilic uptake and phagocytosis of *S. pneumoniae* was shown to be negatively impacted by a preceding viral infection, which can be attributed to the elevated attachment of *S. pneumoniae* to particular cell types via influenza virus-mediated exposure of surface receptors [85]. So-called ‘primary/azurophil granules’, that come into contact with phagocytosed pathogens by fusing with the phagosome, mediate intracellular killing. These granules are characterized by the presence of myeloperoxidase (MPO) and contain anti-bacterial agents [86]. MPO concentrations were shown to be decreased in response to previous influenza virus infection, which hampers intracellular killing of *S. pneumoniae* and the process of phagocytosis [87]. In addition to the primary/azurophil granules, the so-called ‘respiratory burst’ is another signaling process used by neutrophils to kill phagocytized pathogens. Reactive Oxygen Species (ROS) are crucial for this defense mechanism [88]. Strikingly, an influenza virus infection three days prior to *S. pneumoniae* infection was shown to reduce the ability of lung-recruited neutrophils to generate ROS in response to a bacterial challenge in mice, thereby harming the intracellular killing ability of these cells. The same failure in nicotinamide adenine dinucleotide phosphate oxidase-dependent ROS production was described in a case of influenza virus infection six days prior to a secondary bacterial infection. However, in the latter co-infection scenario, this effect was shown to be systemic and included bone-marrow-derived neutrophils [85]. In line with this result, opsonophagocytic killing by neutrophils was demonstrated to be reduced by a preceding influenza virus infection [87]. Nevertheless, the notion of a systemic influence of influenza virus on bone-marrow-derived neutrophils is controversial and the specific alterations induced on the molecular level remain elusive [82,87].

As mentioned above, co-infections can dysregulate the activity of phagocytic cells towards secondary bacterial infections. Specifically, the ability of *S. aureus* to hide intracellularly from the detection of immune cells [89] makes clearance by phagocytes difficult [90]. Thus, the influence of co-infections on immune cells of the innate immune response is a broad field that requires further investigation.

### 2.2. Adaptive Immunity

#### 2.2.1. T Cells and Their Cytokine Responses

The class of T cells includes several different subsets. The most abundant types are CD4+ and CD8+ T cells, also called T-helper cells (Th) and cytotoxic T lymphocytes (CTL), respectively, which are activated by antigen-presenting cells (APCs). In the case of a viral or bacterial infection in the lung, respiratory dendritic cells (DCs) can act as APCs [91]. Depending on the cytokine profile, activated CD4+ cells are further divided into several subgroups, such as Th1 cells (involved in cellular immune responses), Th2 cells (involved in the humoral immune response) and Th17 cells (involved in inflammatory processes) [92]. The development and maintenance of Th17 cells is driven by APC-expressed IL-23. Th17 cells are major producers of the cytokines IL-17 and IL-22 [72]. As mentioned above, these cytokines are connected to neutrophil recruitment and support B cell functions, which is important for pathogen clearance. Influenza virus-induced type I IFN signaling was shown to result in decreased expression levels of IL-23 by DCs. Thus, a preceding influenza virus infection negatively influences the Th17 cell-mediated anti-bacterial response by disrupting signaling between APCs and Th17 cells [72]. The fact that influenza viruses failed to suppress IL-23 production in *Ifnar1*^−/−^ mice confirmed the proposed IFN-dependent mechanisms. Furthermore, adenovirus-induced overexpression of IL-23 in co-infected mice resulted in increased IL-17 and IL-22 levels, accompanied by more efficient bacterial clearance [72]. In contrast to the T cells mentioned above, which display a T cell receptor (TCR) composed of an α- and β- subunit, the TCR of γδ T cells is composed of a γ- and δ- subunit. Even though γδ T cells only make up a small percentage of T cells, it is important to mention that they are not dependent on MHCs, but are capable of direct interaction with an antigen, such as the influenza virus proteins matrix protein 1, nucleoprotein, polymerase acidic protein, polymerase basic protein 2 and HA [93], eventually inducing cell lysis of infected cells [94]. Type I IFN expression induced by influenza virus infection also inhibits IL-17 expression by γδ T cells in co-infections with *S. pneumoniae*. This influenza virus-mediated change in T cell signaling was shown to be connected to diminished bacterial clearance. Accordingly, the transfer of γδ T cells from *Ifnar1*^−/−^ to WT mice resulted in decreased susceptibility to secondary bacterial infections [71].

In addition to the reduced CD4+-mediated bacterial clearance, killing of virus infected cells via CTLs was shown to be affected in co-infections of mice with *S. pneumoniae* and influenza virus. This was caused by a diminished amount of virus-specific CTLs, accompanied by reduced TNFα and IFNγ production, additionally pointing to dysregulated cytokine production. Furthermore, this effect was accompanied by a significant increase in the number of regulatory T cells (Tregs) in the lung [95]. In general, Tregs comprise a specialized T cell subgroup, which suppresses the activation of the immune response to regulate self-tolerance of the immune system [93]. The exact effect of the aforementioned observation on the T cell response in co-infections is not fully understood.

As already mentioned above, *S. aureus* is known for the induction of a massive secretion of inflammatory cytokines and chemokines. Secreted bacterial superantigens induce an uncontrolled polyclonal activation of T cells by cross-linking the MHCII molecules with TCRs, resulting in severe illnesses, including toxic shock syndrome and lung tissue disruption. However, the molecular mechanisms of cross-linking are not entirely clear [28].

In conclusion, T cell activation can be differentially affected by influenza virus and *S. pneumoniae* or *S. aureus* co-infections. Altered secretion of distinct cytokines and chemokines upstream and downstream of T cell activation can have an impact on bacterial as well as viral clearance and, in terms of elevated pro-inflammatory cytokine production in *S. aureus* co-infections, can lead to a cytokine storm, associated with massive lung damage and severe disease progression.

#### 2.2.2. B Cells and Antibody Production

B cells are the only type of cells that are able to differentiate into plasma cells and therefore produce antibodies. In general, APCs engulf viral or bacterial proteins and convert them into immune peptides, which are presented to CD4+ cells. CD4+-polarized cells bind to the MHCII-presented immune peptides via the TCR and ensure the polarization of B cells and their maturation into plasma cells [96].

In influenza virus infections, the lymph nodes in the lung are the main sites of B cell activation. In this regard, the stimulation of B cells was shown to be directly activated via influenza virus-induced type I IFNs [97]. Viral antigen presentation results in the rapid formation of a strong and quick B cell proliferation into short-lived plasma blasts that secrete immunoglobulin M (IgM), immunoglobulin A (IgA) and immunoglobulin G (IgG) [98]. IgA and IgM are present in body secretions and build the first defense mechanisms against pathogens, which can then be neutralized or opsonized by the complement system or the Fc receptors on DCs, macrophages and B cells. Afterwards, so-called germinal center-derived plasma cells are formed when the infection has almost subsided. This immunological memory produces long-lived plasma cells that secrete antibodies (IgG) through a variety of signaling cascades [29]. For *S. aureus* and *S. pneumoniae* infections, the classical pathway of the complement system can additionally build a bridge to adaptive immunity. Opsonization through C3 can lead to the activation of B cells, mediated by the complement receptors CD21 and CD35. This recognition leads to the activation of further signal transduction pathways to form IgM memory B cells without the interaction of T cells. Later on, these IgM memory B cells can further differentiate into staphylococcal- or pneumococcal-specific IgA- or IgG-producing memory B cells or plasma cells [99,100,101]. Compared to influenza viruses, bacteria are more complex and have the ability to secrete different molecules that are described to impair B cell activation or maturation. Regarding *S. aureus*, the secretion of these virulence factors is regulated via the *S. aureus* exoprotein expression two-component system and *agr* [102]. To date, it is not known which factors are responsible for the decreased association of human B cells with antigens on the surface of *S. aureus,* thereby impairing B cell-mediated anti-bacterial activation.

**Figure 3:**
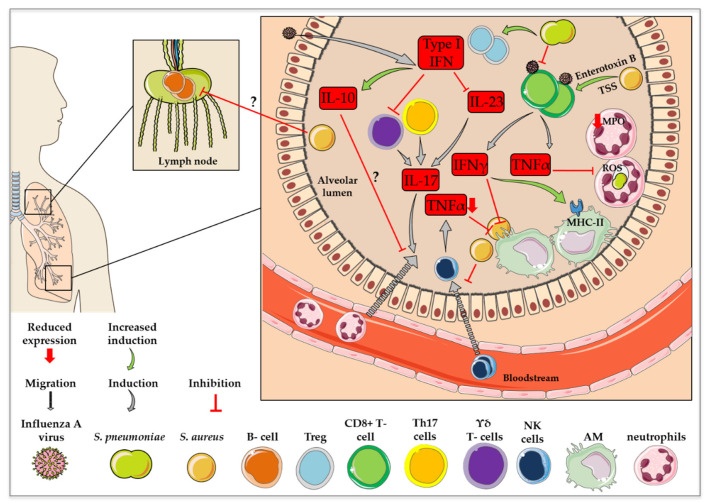
Changes in immune cell recruitment and activation in influenza virus co-infection with *S. pneumoniae* or *S. aureus*. This figure was created using Servier Medical Art templates, which are licensed under a Creative Commons Attribution 3.0 Unported License; https://smart.servier.com (accessed on 11 February 2021).

In the case of a co-infection of mice with influenza virus and *S. pneumoniae*, not only was the co-presence of the pathogens decisive for the activation of B cells, the order of pathogen exposure was also relevant [103]. Primary *S. pneumoniae* with subsequent influenza virus infection led to increased B cell reactions in the lymph nodes and spleen, as well as elevated amounts of CD4+ T follicular helper cells without the induction of a long-lasting immune cell memory. In contrast, primary influenza virus with subsequent *S. pneumoniae* infection resulted in an exaggeration of CD4+ T cell activation, which led to long-lasting titers of anti-viral serum IgGs with improved virus neutralizing activity [103]. In the case of the intracellular occurrence of the pathogens influenza virus and *S. aureus*, APCs phagocytose the infected cells. Intracellular viral or bacterial components are then presented on the surface of the macrophages via MHCII [104]. For instance, mice commensally colonized with *S. aureus* were protected from subsequent lethal influenza virus infections [105] due to upregulated antigen presentation by MHCII, with possible elevated B cell activation [106]. Figure 3 presents a summary of the impact of influenza virus co-infection with *S. pneumoniae* or *S. aureus* on immune cell recruitment and activation.

In summary, further investigations regarding the role of B cells in co-infections with influenza virus and *S. pneumoniae* or *S. aureus* are necessary, since only little is known about the interaction of the pathogens with B cells. Nevertheless, B cell activity can be affected differently by each of the pathogens. Additionally, the order of pathogen exposure in co-infections might have an influence on the development of long-lasting B cell memory.

## 3. Conclusions

Even though co-infections with influenza viruses and *S. pneumoniae* or *S. aureus* have been known to exist for decades, they still present a challenge in clinics. Therefore, it is of major importance to obtain a better understanding of the complex interactions of the pathogens with the host and with each other, in order to potentially identify new targets for therapeutic interventions to mitigate or even impede secondary bacterial infections in the future.

Here, we have summarized the current knowledge regarding innate and adaptive immunity during co-infections, including information on specific signal transduction pathways, which take part in the establishment of appropriate immune responses. In comparison to single infections, the presence of both pathogens results in dysregulated cytokine and chemokine production after pathogen sensing via typical PRRs, leading to dynamic changes in immune cell recruitment and activation. On the one hand, hyper-activated signaling pathways result in a massive recruitment of immune cells and an overshooting of inflammatory processes, leading to severe lung damage. Subsequently, this massive tissue injury enables the deeper penetration of bacterial pathogens into the tissue and allows uncontrolled replication of the viral and bacterial pathogens. On the other hand, a weakened immune response through the inhibition of immune cells or signaling cascades by the pathogens leads to suppressed immune reactions, resulting in lowered pathogen clearance. This ultimately leads to pathogen-beneficial outcomes through an imbalanced immune response, with increased morbidity and mortality rates.

## Figures and Tables

**Table 1:** List of cytokines and chemokines induced and their roles in influenza virus and *S. pneumoniae* or *S. aureus* co-infection.

Cytokines/ Chemokines	Function in Co-Infection	Reference
	**Innate**	
IFNα/IFNβ	Abolishment of bacterial clearance	[45]
IL-1β	Exaggeration of the immune response	[70]
IL-6	Increased expression of acute-phase proteins and mediation of cell homeostasis and phagocytosis	[54,56]
IL-10	Inhibition of neutrophil recruitment	[49]
RANTES	Recruitment of neutrophils	[68]
IP-10 MCP-1 MIP1/MIP2	Massive recruitment of macrophages and uncontrolled immune cell activation	[66,67]
	**Adaptive**	
IL-17	Bacterial clearance	[71]
IL-23	*S. aureus* clearance	[72]
IFNγ	Uncontrolled T cell activation	[64]
TNFα	Exaggeration of the immune response, impairment of alveolar macrophages’ phagocytic activity	[70,73]
